# Whither Inhibition?

**DOI:** 10.1177/09637214221095848

**Published:** 2022-07-14

**Authors:** Kaitlyn M. Werner, Michael Inzlicht, Brett Q. Ford

**Affiliations:** 1 Department of Psychology, University of Toronto; 2 Department of Psychology, University of Pennsylvania; 3 Rotman School of Management, University of Toronto

**Keywords:** inhibition, goal pursuit, strategies, process model, cognitive control, executive function, willpower

## Abstract

Inhibition is considered a process essential to goal pursuit and as a result has become a central construct in many disciplines in psychology and adjacent fields. Despite a century’s worth of debate, however, there is little consensus about what inhibition actually is. We suggest that it is time to abandon the concept of inhibition as it currently stands, given that its definition has been problematic. Instead, we propose an alternative framework in which inhibition is the target outcome, rather than a process to obtain a goal. We leverage existing process models to elucidate how people can achieve an inhibition goal by actively regulating impulses and desires. Although the field has been led astray by classifying inhibition as a process, our framework is intended to provide greater practical utility to the study of goal pursuit moving forward.

Inhibiting mental processes is considered essential for successful goal pursuit, whether the goals relate to basic motor movements, thoughts and emotions, or higher-order objectives (e.g., health, relationships, finances). The concept of inhibition has deep roots, tracing back to ancient philosophers as well as to the father of modern psychology, William James, who stated that inhibition is “an essential and unremitting element of our cerebral life” (James, 1890, p. 583). Although inhibition seemingly plays a key role in many areas of psychology (e.g., cognitive, social, personality, developmental, and clinical psychology) and adjacent fields (e.g., neuroscience and economics), there is little consensus about what inhibition actually is. For more than a century, researchers have debated the nature of inhibition (Breese, 1899; Harnishfeger, 1995; MacLeod et al., 2003), but there is no resolution in sight.

In this article, we suggest that it is time to abandon the concept of inhibition as it currently stands. To advance research on inhibition, we first provide an overview of what is problematic about the concept of inhibition. To address these concerns, we then present an alternative framework that reclassifies inhibition as an outcome rather than a process, thereby providing greater conceptual and practical utility to inhibition as a construct. Finally, we close by outlining promising directions for future research.

## Fundamental Issues With Inhibition

Over the years, scholars have raised numerous concerns about inhibition. Here, we consider three interrelated issues: (a) Different subfields disagree on how to define inhibition, which has led to a proliferation of different operationalizations; (b) many areas have seemingly lowered the threshold for what counts as inhibition, diluting its meaning as a construct; and (c) the term “inhibition” has been overextended to concepts that can be more parsimoniously explained by other constructs. It is important to note that we make few exceptions to these concerns, the most notable being in regard to neural inhibition: It is widely accepted that, from a biological standpoint, neurons can inhibit each other (MacLeod et al., 2003). Here, we focus solely on contexts in which the term “inhibition” is used to describe the downregulation of mental and behavioral processes, in which case the process at the neural level tends to be biologically excitatory.

### Proliferation of definitions

One pressing concern is that researchers rarely explicitly define inhibition (MacLeod, 2007), and when definitions are provided, they are often broad and/or differentially operationalized across disciplines. For example, inhibition has been broadly defined as “any mechanism that reduces or dampens neuronal, mental, or behavioral activity” (Clark, 1996, p. 128), but also as a cautious temperament during development (Tang et al., 2020). Some researchers draw more nuanced distinctions by focusing on specific types of inhibition, which has spurred a long-standing debate on whether inhibition is a single general construct that applies across contexts or a series of unique processes that operate in different contexts (e.g., behavioral inhibition, cognitive inhibition, neural inhibition; Harnishfeger, 1995). To date, it seems that the only thing researchers know for sure about inhibition is that there is no consistent definition of what it is. Although having a far interdisciplinary reach should promote cumulative science (Lin et al., 2021), it appears that the interdisciplinary nature of inhibition has led to a fragmented field, which has caused theoretical, practical, and methodological advancement to stagnate.

### Dilution of the construct

As is the case with many concepts in psychology, inhibition has undergone many semantic and conceptual shifts over time. As a result of this concept creep (conceptual expansion; Haslam, 2016), inhibition encompasses a much broader range of phenomena than ever before. Some expansion is beneficial, such as when a concept is broadened outward to new contexts; for example, the concept of bullying has been rightfully expanded to new contexts, such as online behavior (cyberbullying) and certain types of social exclusion in which the focus is on harmful omission rather than direct hurtful actions toward the victim (Haslam, 2016). However, other types of expansion are more problematic, such as when the threshold for what counts as an instance of a phenomenon becomes less stringent. For example, writing a one-off angry social-media post about your classmate after a bad day may in certain situations be improperly perceived as cyberbullying. The concept of inhibition has indeed been overextended to the “point of meaninglessness” (Aron, 2007, p. 219). This is likely because what counts as inhibition is largely subjective, which has allowed researchers to easily classify any behavior as inhibition. For example, people who choose pizza over salad are often assumed to have failed to inhibit their desire for pizza, yet it is equally plausible that no conflict requiring inhibition existed in the first place (e.g., they may have budgeted the treat into their daily calories). Although concept creep is inevitable among popular constructs, such as inhibition, it is imperative that researchers work together to monitor how concepts evolve and to decide whether a particular trend should be encouraged, ignored, or resisted (Haslam, 2016).

### Overextension to other constructs

Because of its ever-expanding nature, the construct of inhibition has become overextended, which has limited its conceptual and practical utility. However, this overextension is not merely a product of a lowered threshold; there are notable instances of some phenomena being incorrectly labeled as inhibition. For example, the color-word Stroop task, one of the most popular measures of inhibition, was not actually designed to test inhibition. It is actually a measure of interference (i.e., decreased performance caused by irrelevant information; Stroop, 1935), and someone can be successful without needing to “inhibit” word reading (Cohen, 2017; Cohen et al., 1990). Thus, the outcomes of this task can be more aptly explained by other processes, such as selection of some stimuli for engagement over others^1^ or automatic memory retrieval (Cohen et al., 1990; MacLeod et al., 2003). Similar ambiguities exist in memory research. Inhibition was originally proposed to be a process underlying retrieval-induced forgetting (Anderson & Spellman, 1995). However, more contemporary views now cast doubt on inhibition’s role, suggesting that retrieval-induced forgetting is best explained by memory retrieval (see MacLeod et al., 2003). A final example is the behavioral inhibition system, a construct popular within personality psychology. Defined as an individual’s tendency to inhibit behavior leading to aversive outcomes (Carver & White, 1994), this construct overlaps highly with neuroticism and social anxiety, which suggests that it likely reflects emotional instability rather than ability to inhibit unwanted responses.

### Interim summary

Taken together, these issues of proliferation, dilution, and overextension have undermined research on inhibition. Despite the long-standing debate, there has been no resolution regarding how to define inhibition, let alone operationalize it. This raises the question: Why are researchers still holding on to the idea that inhibition is a process? We suggest that operationalizing inhibition as a process has been hard to let go because there has yet to be a viable alternative framework for studying the actual processes through which people control their impulses and desires.

## A Framework for Examining Inhibition in the Context of Goal Pursuit

To move toward a productive next generation of research, we suggest, it is time to abandon the concept of inhibition as it currently stands, given that its definition so far has been problematic. Instead, research can refocus on the process of goal pursuit in order to provide a better understanding of how people can achieve their goals. Research examining people’s use of inhibition tells little about the processes people use to regulate impulses and desires, so articulating a framework that will provide better understanding of these processes is critical for helping people pursue their goals.

### Inhibition is the outcome, not the process

We propose that conceptual clarity has been elusive because inhibition has been inaccurately operationalized as a process to obtain a goal (i.e., a mechanism that creates the outcome), when in fact inhibition is the goal itself. People do not *use* inhibition to suppress a target response; rather, the *goal* is to inhibit the target response, and to do this successfully, people must rely on other processes. Thus, we define inhibition as the goal of stopping a mental, behavioral, or emotional response (e.g., stopping oneself from eating a cupcake, expressing one’s anger, or pressing a designated key).

Consider an illustrative example. The stop-signal task is a cognitive reaction time task in which a person is asked to respond quickly to a target stimulus (e.g., a square) but inhibit the response when presented with a designated stop signal (e.g., an auditory sound) that appears after a short delay period. Thus, the goal is to stop one’s response on trials that randomly include the delayed stop signal. In this context, inhibition is not a process to be “used” to downregulate a motor response; rather, inhibition is the goal of stopping a motor response, and this goal can be achieved in a number of ways (e.g., proactive strategies; Braver, 2012). For example, increased mental preparation in the stop-signal task results in more efficient inhibition, a finding that has been replicated using behavioral and neuroimaging measures (e.g., reaction time and activation of frontoparietal regions, respectively; Chikazoe et al., 2009). Another example is the goal to inhibit one’s desire for tempting unhealthy foods. Research shows that when people are asked to inhibit their desire for unhealthy food, they actually engage in other processes that they misattribute to inhibition (e.g., distracting themselves, thinking about unappealing qualities of the food; Werner et al., 2021). Together, these findings highlight that people do not use inhibition to achieve a desired outcome; rather, inhibiting a target response is the desired outcome, and people use a variety of processes to successfully achieve this goal.

### Leveraging process models to facilitate goal attainment

To better understand goal pursuit, researchers need to shift their focus away from inhibition as a process and instead toward the actual processes that help people reach their goals of inhibiting impulses and desires. To do this, researchers can leverage existing process models to more precisely identify the processes that are effective in reaching these goals, ultimately providing greater practical utility to the study of inhibition. To help guide future research and theorizing on inhibition, we highlight a recent process model that describes goal pursuit as a dynamic, multistage process, beginning with identifying which goals to pursue and continuing with selecting strategies, implementing those strategies, and subsequently monitoring each of these processes over time in order to reach the target goal (Duckworth et al., 2016; Gross, 2015; Werner & Ford, 2021).

**Fig. 1.**
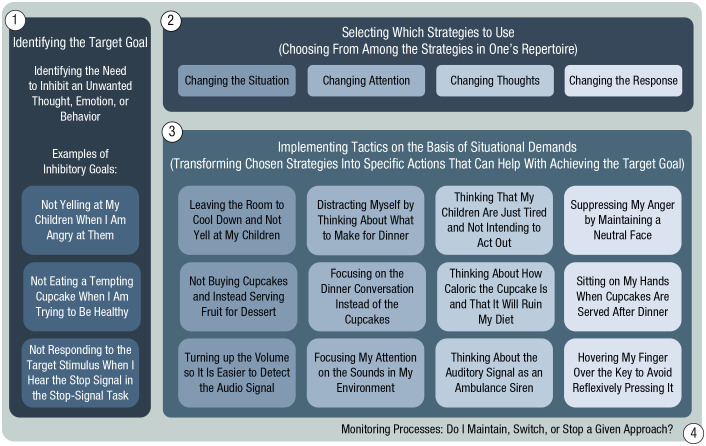
A process model with inhibition as the goal (adapted from Gross, 2015; Werner & Ford, 2021). First, one identifies the target goal, in this case, to inhibit an unwanted thought, emotion, or behavior. Second, one selects which strategies to use on the basis of the strategies available in one’s repertoire (i.e., one’s regulatory toolbox). These strategies include changing the situation (i.e., modifying some aspect of the environment), redirecting attention (i.e., changing the focus of one’s attention), changing thoughts (i.e., changing how one is thinking about the situation), and changing the response (i.e., changing one’s experiential, behavioral, or physical response). Third, one implements tactics by transforming the chosen strategies into specific actions. Fourth, and finally, one actively monitors all processes in order to decide whether to maintain, switch, or stop the approach (i.e., the goal, strategy, or tactic) in view of whether or not the inhibition goal has been achieved. For example, if a particular strategy or tactic has not been successful (e.g., the desire for the cupcake still persists), one may opt to change strategies or tactics in order to successfully inhibit the target response.

The process model of emotion regulation (Gross, 2015) is arguably one of the most influential models within psychology, and it has been adapted to relevant contexts, including self-control (Duckworth et al., 2016; Werner & Ford, 2021) and behavior change (Duckworth & Gross, 2020). The most recent adaptation, the extended process model of self-control (Werner & Ford, 2021), can be readily applied to inhibition (see Fig. 1): A person identifies the goal to inhibit a target response, such as the goal to inhibit a negative emotional response when angry (e.g., “do not yell at my kids”), inhibit one’s desire for an unhealthy, yet tasty treat (e.g., “do not eat the cupcake”), or inhibit a target response during a computerized cognitive task (e.g., “do not press the button on trials with a sound”).

Once a goal is set, the next step is to select strategies that can be used to reach the desired outcome. Strategies can be used at different points in the response cycle. To reach their inhibition goal, people can change the situation, change what they are paying attention to, change how they think about the situation, or change their behavioral and/or physiological response. Moreover, they can flexibly use these strategies in ways that best match the situational demands (Bonanno & Burton, 2013; Werner & Ford, 2021).

Once a strategy (or strategies) is chosen, it is then implemented using specific tactics that are most effective in the moment, such as leaving the room when angry in order to be alone and cool down for a bit (i.e., situation modification), thinking about the negative consequences of eating a tempting food (i.e., cognitive reappraisal), or intensely focusing attention on the computer screen while waiting for the auditory signal in the stop-signal task (i.e., attentional deployment). If the tactic is successful (i.e., one does not react in anger, resists eating the food, or withholds the key press at the appropriate time), the inhibition goal is successfully attained. Finally, the progress at each stage is actively monitored so that one can maintain, stop, or switch from a particular process when necessary.

### Why does the process-versus-outcome distinction matter?

Conceptualizing inhibition as an outcome rather than a process has many important implications for the study of goal pursuit. Rightfully operationalizing inhibition as a goal allows the field to better focus on the actual processes people use to pursue their goals. As a result, researchers not only are able to determine which strategies are actually most effective, but also can focus on important questions, including how personal factors can influence the processes people use (who uses what processes and when?), how contextual factors influence the effectiveness of different processes (when are particular processes most effective in the short run?), and whether certain processes are more adaptive than others (what are the most adaptive patterns of processes people can use to promote long-term success? e.g., Ford & Troy, 2019; Werner & Ford, 2021).

This shift to inhibition being a goal also has important methodological implications. Researchers typically use an “inhibition” condition as the gold-standard comparison condition when trying to understand the effectiveness of different processes (e.g., cognitive change). However, comparing processes with inhibition is methodologically problematic: Because inhibition is not a process, telling people to “use inhibition” actually assigns them the goal to inhibit the target response. They then rely on whatever processes they have at their disposal, which creates noisy variability (e.g., when “using inhibition,” some people rely on distraction, and others rely on cognitive change; Werner et al., 2021). This variability makes it difficult (if not impossible) to determine the true effectiveness of the comparison processes. Thus, future research would greatly benefit from examining the specific processes through which people can attain their goals.

## Directions for Future Research

Adopting a process-oriented framework has important implications for multiple subfields within psychology and adjacent fields. Here, we highlight three examples, outlining promising directions for future research.

### Social and personality psychology

In social and personality psychology, inhibition has been commonly known as *willpower* (Baumeister et al., 2007). Traditionally, research has promoted the benefits of willpower, although recently researchers have questioned its validity as a strategy (Werner et al., 2021), likening telling people to “use willpower” to telling them “to build a house with a pile of wood” (Fujita et al., 2020, p. 150; see also Gross & Duckworth, 2021). Common operationalizations of willpower include assigning participants the goal to inhibit a target response and assessing individual differences in ability to make progress on an inhibition goal; however, neither operationalization reveals the underlying processes that help people achieve their goals. Moving beyond willpower, researchers can instead examine the strategies people use to inhibit unwanted desires, including how people choose what strategies they use (including whether they choose a single strategy or multiple strategies), the flexibility with which they use strategies in different contexts, and when and for whom each strategy is most adaptive (Bonanno & Burton, 2013; Werner & Ford, 2021).

### Cognitive psychology

In cognitive psychology, inhibition is associated with research on cognitive control (also called executive function), which refers to the ability to pursue goal-directed behavior, even in the face of more habitual or immediately compelling behaviors (Cohen, 2017). Although inhibition is considered one of the core processes that power cognitive control (Miyake et al., 2000), there is little direct evidence that inhibition is actually needed to implement cognitive control (Cohen, 2017). For example, Stroop and stop-signal performance can be improved by selectively attending to task instructions (to name colors) and mentally preparing (to proactively attend to the stop signal); good performance on these tasks does not necessarily require reactively stopping a competing mental process. Thus, although performance on such tasks loads onto the same factor (i.e., performance on these tasks is highly correlated and therefore taps into the same abilities; Miyake et al., 2000), the name of this factor need not be “inhibition.” Indeed, other analyses suggest that the so-called inhibition factor is indistinguishable from a factor that taps the general speed of processing (Jewsbury et al., 2016) or even a factor that is common to all executive-function tasks (Friedman & Miyake, 2004). In other words, with the possible exception of stopping motor commands (Aron, 2007), inhibition might be unimportant in the control of attention. Instead, what has previously been bundled under the “inhibition” label might simply reflect the ability to efficiently select some processes for engagement over others, a key feature of both cognitive control and intelligence (Jewsbury et al., 2016). Expanding on these findings, future research should begin to focus more on comparing the effectiveness of specific cognitive processes that people use to inhibit their responses (Chikazoe et al., 2009).

### Clinical psychology

Within clinical psychology, various forms of psychopathology are characterized by the inability to inhibit responses appropriately. Indeed, not being able to inhibit responses is associated with a range of negative mental-health outcomes, including depression, attention-deficit/hyperactivity disorder, bipolar disorder, and substance abuse (Wright et al., 2014). One attempted solution has been to use inhibition training (i.e., teaching people to repeatedly stop their response to a particular cue) to reduce problematic behaviors (e.g., alcohol consumption, gambling, overeating); however, research suggests that such training does not offer any appreciable effects (Kassai et al., 2019), possibly because (at least in part) training people to “use inhibition” is so vague that it cannot transfer to real-world behavior. Given the futility of such training, researchers should instead focus on unpacking the underlying processes that explain why a person cannot adaptively inhibit target outcomes (Sheppes et al., 2014). For example, one hallmark of people suffering with negative mental health is their rigid and limited use of coping strategies. To help, researchers can develop interventions that provide patients with a toolbox of strategies that they can then flexibly choose from in different situations (cf. Southward et al., 2021).

## Conclusion

For centuries, the concept of inhibition has played a prevalent role in understanding human functioning. Given the many conceptual issues surrounding inhibition, we propose that it is time to abandon this concept as it currently stands. We suggest treating inhibition as an outcome and turning researchers’ attention to studying the actual processes through which people achieve their goals. Our hope is that reframing inhibition this way will push the field toward greater conceptual and empirical clarity. Finally, inhibition is merely one of many constructs in psychology (and adjacent fields) that have fallen victim to overexpansion and are now conceptually ambiguous. Although our aim here has been to help clarify the concept of inhibition specifically, we hope that this work inspires other researchers to tackle these issues within their respective disciplines.

## Recommended Reading

Gross, J. J. (2015). (See References). A detailed overview of the process model of emotion regulation (which can also be applied to self-regulation more broadly), including how people identify which regulatory goals to pursue, select strategies for pursuing them, and subsequently implement those strategies.

Inzlicht, M., Werner, K. M., Briskin, J. L., & Roberts, B. W. (2021). Integrating models of self-regulation. *Annual Review of Psychology, 72*, 319–345. https://doi.org/10.1146/annurev-psych-061020-105721. A comprehensive overview that integrates several models of self-regulation from different subfields (e.g., social and personality psychology, cognitive neuroscience) and highlights their points of convergence and divergence on important components of regulation, including conflict, emotion, and cognitive functioning.

MacLeod, C. M., Dodd, M. D., Sheard, E. D., Wilson, D. E., & Bibi, U. (2003). (See References). A “reader’s digest” history of inhibition, including a variety of case studies highlighting how the concept of inhibition has been used in different areas in cognitive science, as well as some of the definitional issues surrounding inhibition.

Werner, K. M., & Ford, B. Q. (2021). (See References). An application of the process model of emotion regulation to self-control, accompanied by discussion of the importance of having a well-equipped strategy toolbox and engaging in regulatory flexibility (i.e., choosing strategies appropriate to a particular context), as well as polyregulation (e.g., pursuing multiple goals, using multiple strategies).

## Transparency

*Action Editor:* Robert L. Goldstone

*Editor:* Robert L. Goldstone

## Notes

1. It has also been proposed that Stroop performance can be better explained by attention, but attention is also composed of its own set of processes that are up for debate. We do not want to replace one ambiguous concept with another, and instead we merely state that cognitive effects thought to reflect inhibition can often be explained without recourse to inhibitory processes.
